# Prognostic Modeling of COVID-19 Using Artificial Intelligence in the United Kingdom: Model Development and Validation

**DOI:** 10.2196/20259

**Published:** 2020-08-25

**Authors:** Ahmed Abdulaal, Aatish Patel, Esmita Charani, Sarah Denny, Nabeela Mughal, Luke Moore

**Affiliations:** 1 Chelsea and Westminster NHS Foundation Trust London United Kingdom; 2 NIHR Health Protection Research Unit in Healthcare Associated Infections and Antimicrobial Resistance Imperial College London London United Kingdom

**Keywords:** COVID-19, coronavirus, machine learning, deep learning, modeling, artificial intelligence, neural network, prediction

## Abstract

**Background:**

The current severe acute respiratory syndrome coronavirus 2 (SARS-CoV-2) outbreak is a public health emergency and the case fatality rate in the United Kingdom is significant. Although there appear to be several early predictors of outcome, there are no currently validated prognostic models or scoring systems applicable specifically to patients with confirmed SARS-CoV-2.

**Objective:**

We aim to create a point-of-admission mortality risk scoring system using an artificial neural network (ANN).

**Methods:**

We present an ANN that can provide a patient-specific, point-of-admission mortality risk prediction to inform clinical management decisions at the earliest opportunity. The ANN analyzes a set of patient features including demographics, comorbidities, smoking history, and presenting symptoms and predicts patient-specific mortality risk during the current hospital admission. The model was trained and validated on data extracted from 398 patients admitted to hospital with a positive real-time reverse transcription polymerase chain reaction (RT-PCR) test for SARS-CoV-2.

**Results:**

Patient-specific mortality was predicted with 86.25% accuracy, with a sensitivity of 87.50% (95% CI 61.65%-98.45%) and specificity of 85.94% (95% CI 74.98%-93.36%). The positive predictive value was 60.87% (95% CI 45.23%-74.56%), and the negative predictive value was 96.49% (95% CI 88.23%-99.02%). The area under the receiver operating characteristic curve was 90.12%.

**Conclusions:**

This analysis demonstrates an adaptive ANN trained on data at a single site, which demonstrates the early utility of deep learning approaches in a rapidly evolving pandemic with no established or validated prognostic scoring systems.

## Introduction

Since the outbreak of severe acute respiratory syndrome coronavirus 2 (SARS-CoV-2) in Wuhan, China in December 2019, there have been over 229,705 confirmed cases in the United Kingdom, with a case fatality rate of 14.4% as of May 13, 2020 [1,2]. In the United Kingdom, the highest number of coronavirus disease (COVID-19) deaths (the disease caused by the SARS-CoV-2 virus) has been reported in London [3], with many health care providers having experienced a rapid, difficult-to-predict increase in intensive therapy unit (ITU) bed requirements.

Although there appear to be several early predictors of outcome such as age, high sequential organ failure assessment score and elevated D-dimer levels [4], being male [5], poor glycemic control in patients with diabetes [6], being immunocompromised [7], and obesity [8], currently there are no validated prognostic models or scoring systems applicable specifically to patients with SARS-CoV-2, despite attempts to delineate general predictors of mortality [9]. Emerging clinical risk scores have been limited by small sample sizes [10], predicting outcomes in suspected as well as confirmed cases [11-14], or using regression analysis to produce static models that have been applied to specific population subgroups, limiting generalizability [15].

Predicting patient-specific adverse events including ITU admissions and mortality with sufficient lead time is crucial during a pandemic, as it allows clinicians, managers, and service providers to admit patients based on risk of deterioration, forecast ITU bed demand, and determine appropriate ceilings of care. From a public health perspective, this would play a significant role in allowing policy makers to respond efficiently to surges of COVID-19, which would otherwise risk overwhelming critical care capacity [16].

There have been significant recent advances in modeling clinical data on electronic health records (EHRs) [17,18] and specifically in the capability of machine learning techniques to predict mortality [19-21]. In view of this, our study proposes an artificial neural network (ANN) that analyzes a set of patient features including demographics, comorbidities, lifestyle factors, and presenting symptoms and predicts patient-specific mortality risk during the current hospital admission. Crucially, this data could be collected during the initial encounter of a patient with a physician, and therefore allows for a prediction of outcome at the earliest opportunity along the patient pathway.

Classically, deep learning approaches created models that were difficult to interpret. This had led clinicians to retreat from complex but accurate techniques to simpler (eg, linear) models [22]. However, significant recent advances have been made in deep learning interpretability research [23] and specifically in creating predictive machine learning models for health care [24-26]. We use an algorithm capable of revealing which features were important for making predictions while maintaining accuracy and consistency [27].

## Methods

The ANN was trained on retrospective data extracted from EHRs in a digital format. Demographic, comorbidity, lifestyle, and symptom data were encoded from admission notes of patients admitted to an accident and emergency department at a West London teaching hospital.

### Study Population and Data Description

The clinical data used in this study was collected from all hospital admissions for SARS-CoV-2 from February 2, 2020, to April 22, 2020, at a West London teaching hospital. All patients were included in the analyses. Data was anonymized at point of extraction from EHR software (Millennium, Cerner Corporation) and included admission notes, current active medical conditions, and discharge summaries or electronic certifications of death.

The inclusion criteria included all patients with real-time reverse transcription polymerase chain reaction (RT-PCR) test–confirmed SARS-CoV-2 (proprietary Public Health England Assay until March 10, 2020, and an AusDiagnostics assay thereafter). A SARS-CoV-2 infection must have been the principal diagnosis and the reason for that admission episode. Outcome data including the presence of either a discharge summary or electronic certification of death were collected for each patient. SARS-CoV-2 mortality was defined as an in-patient death, which occurred during the current admission episode, in patients with confirmed SARS-CoV-2.

### Data Preprocessing

Individual patients were represented as an array of possible prognostic factors. Demographic factors included age and gender. Comorbidities included the presence or absence of chronic obstructive pulmonary disease, asthma, or a chronic respiratory disease; hypertension, diabetes, ischemic heart disease, congestive cardiac failure, hepatic cirrhosis, chronic kidney disease, or a cerebrovascular event history. Smoking history was also collected. Symptom data included the number of days of symptoms prior to hospital admission and the presence or absence of fever, cough, dyspnea, myalgia, abdominal pain, diarrhea or vomiting, altered mentation, collapse, and olfactory change or ageusia. These data points represented a feature set that was then used as the input for the ANN. Information regarding patient ethnicity was not felt to be robust due to 23.4% missing data. Ethnic subgroups are reported using descriptive statistics; however, ethnicity is not included in the ANN as a variable.

All data was encoded (by AA, AP, and EC) after reading the admission episode notes for each patient. Comorbidity, lifestyle, and symptom data were encoded as binary presence features. Age was recorded as a discrete quantitative feature. Gender was recorded as a categorical variable, which was later encoded as a numerical binary feature. All numerical features were standardized (centered and scaled) by subtracting the mean and dividing by the standard deviation of the training samples. The target value was defined as an in-patient death in a patient clinically suspected of having SARS-CoV-2 on admission and who had a positive RT-PCR test. Deaths were counted if they occurred during the admission episode for which outcome predictions were made. Mortality was encoded as a binary target value.

### Model for Predicting SARS-CoV-2 Outcomes

#### Overview

The ANN used clinical data accrued from the admission notes to predict mortality for that admission. Input features were provided to the system, and the output is the probability of death for a patient during their current admission. Patients were randomized to training (80%) and testing (20%) sets. Data for each patient were randomized to only one of these sets. To reduce model overfitting, k-fold cross-validation was used. Ten folds were chosen during training, which represented 10% of the training sets being used as validation sets. If the probability of mortality was above a threshold of 50%, the prediction was considered positive in that the model predicted the patient was likely to experience a poor outcome.

#### Artificial Neural Network Input and Core

**Figure 1:**
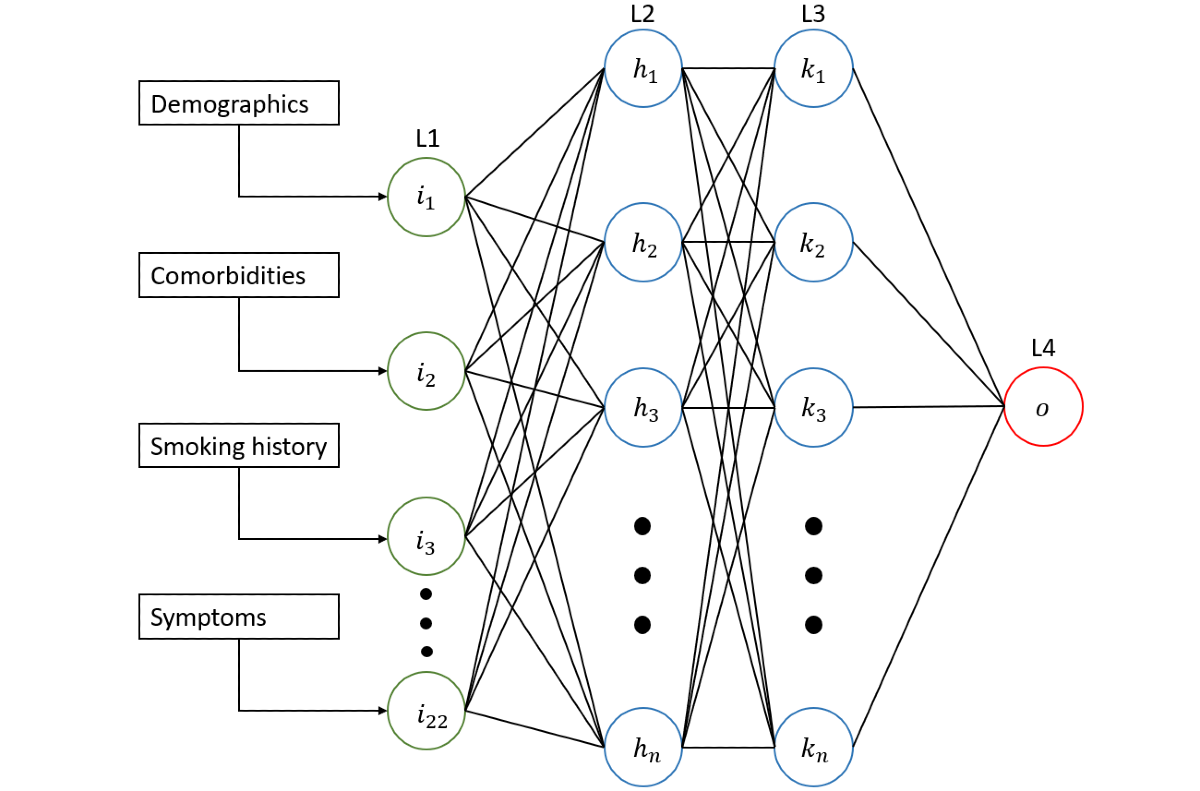
Architecture of an artificial neural network to prognosticate severe acute respiratory syndrome coronavirus 2 (SARS-CoV-2) in a UK population. L1 represents the input layer, which accepts patient features as input. L2 and L3 represent two densely connected hidden layers, with a variable number of units. L4 represents the output layer which predicts mortality for a given patient.

Figure 1 demonstrates the ANN. The input layer had an input dimension equal to the number of patient features and used rectifier activation (n=22). Information was then fed into two densely connected further layers (known as hidden layers), which also used rectifier activation. Hidden layers are input-output transformation of incoming data. Each layer attempts to create increasingly meaningful representations of the input data (patient variables) before attempting to make an outcome prediction.

#### Prediction Targets and Training Objectives

The output layer consisted of a single node which used sigmoid activation to provide a probability for the target value. In other words, the output layer collects the data representations from the prior hidden layers and predicts whether a patient is likely to die during their admission. Each resulting probability output was compared to the ground-truth label using the cross-entropy loss function, which measures the performance of a classification model whose output is a probability value between 0 and 1 [28]. This means that the ANN checks whether the prediction it made was correct against known patient outcomes.

#### Training and Hyperparameters

Model architecture was chosen based on validation set performance. A grid search technique was used to exhaustively search over parameters in an iterative process to establish optimal model parameters (known as hyperparameters). All variables were initialized with normalized uniform (Xavier normal) initialization [29] and trained using the Adam adaptive learning rate optimization algorithm [30]. The input layer had an input dimension to match the number of patient variables (n=22). Optimal validation results were achieved with an ANN with 22 units in the first hidden layer with a dropout rate of 20%, and 6 units in the second and third hidden layers with a dropout rate of 40% to prevent overfitting. To further prevent overfitting, L2 regularization is used on all layers except for the output layer.

#### Relative Importance of Clinical Attributes

We used a high-speed approximation algorithm for Shapley additive explanations (SHAP) values, which in effect reveal the contribution of each patient variable (clinical attribute) to their mortality prediction against a mean prediction [30,31].

### Evaluation of Model

A validation set represented by 10% of the training set was used during cross-validation and a cross-validated grid search. The validation set was used to improve model architecture and select for optimal hyperparameters. The metrics selected for model performance evaluation were accuracy, sensitivity, specificity, positive predictive value, negative predictive value, and the area under the receiver operating characteristic curve (AUROC). K-fold cross-validation allowed a mean model accuracy with 95% CI to be calculated.

### Code Availability

The open source machine learning framework Tensorflow 2.1.0 [32] was used to develop the neural network. The architecture was written in the Python programming language (Python 3.7.7). Scikit-learn 0.22 and its dependencies were used to create the data preprocessing pipeline and to create the graphs in this analysis.

### Ethical Considerations

Data was collected as part of routine care by the responsible clinical team. No patient-identifiable data was used in this analysis. The need for written informed consent was waived by the Research Governance Office of Chelsea & Westminster NHS Foundation Trust. The study protocol was approved by the antimicrobial stewardship group at Chelsea & Westminster NHS Foundation Trust. The study was conducted in accordance with the Helsinki declaration.

## Results

### Patient Demographics, Comorbidities, and Symptoms

There were a total of 11,144 data points for 398 patients, encompassing 22 input features. The training and testing populations consisted of n=318 and n=80 patients, respectively. Out of the 398 patients included in the analysis, 389 (97.8%) had completed outcomes and 9 (2.2%) were still hospitalized at the end of the study period. There were 223 (56%) males. Of patients with completed outcomes, 275 (69%) were discharged alive and 93 (23%) died. There were 53 admissions to the ITU (13%), of which 17 (32%) died.

The median age of all patients was 65 years (IQR 51-80). The median age of patients who were admitted to the ITU was 56 years (IQR 51-65) and the median age of patients who died was 79 years (IQR 72-86). Regarding ethnicity, 157 (39.4%) patients were White, 37 (9.3%) were Black, 42 (10.6%) were Asian, 66 (16.6%) were from other ethnicities, and 3 (0.7%) were of mixed ethnicity. In total, 93 (23.4%) did not have a recorded ethnicity. The median time from symptom onset to hospital admission was 5 days (IQR 2-10). Mean length of stay for all patients with completed outcomes was 9.8 days (SD 9.6). Mean length of stay for patients who died was 8.5 days (SD 7.9).

The most common comorbidities were hypertension (n=147; 37%), diabetes (n=104; 26%), and chronic respiratory disease (n=84; 21%). The most common presenting symptoms were cough (n=247; 62%), dyspnea (n=223; 56%), and fever (n=216; 54%). Tables 1 and 2 demonstrate comorbidities and presenting symptoms in order of prevalence, respectively.

**Table 1:** Prevalence of comorbidities in a severe acute respiratory syndrome coronavirus 2 (SARS-CoV-2) population in West London.

Comorbidity	Patients, n (%)
Hypertension	147 (36.9)
Diabetes	104 (26.1)
Chronic obstructive pulmonary disease, asthma, or chronic respiratory pathology	84 (21.1)
Ischemic heart disease	47 (11.8)
Chronic kidney disease	33 (8.3)
Cerebrovascular event history	29 (7.3)
Cardiac failure	22 (5.5)
Obesity	15 (3.7)
Hepatic cirrhosis	6 (1.5 )

**Table 2:** Prevalence of symptoms in a severe acute respiratory syndrome coronavirus 2 (SARS-CoV-2) population in West London.

Symptom	Patients, n (%)
Cough	247 (62.1)
Dyspnea	223 (56.0)
Fever	216 (54.2)
Diarrhea or vomiting	105 (26.4)
Myalgia	68 (17.1)
Altered mentation	59 (14.8)
Abdominal pain	40 (10.1)
Collapse	37 (9.3)
Anosmia or ageusia	36 (9.0)

### ANN Performance

**Figure 2:**
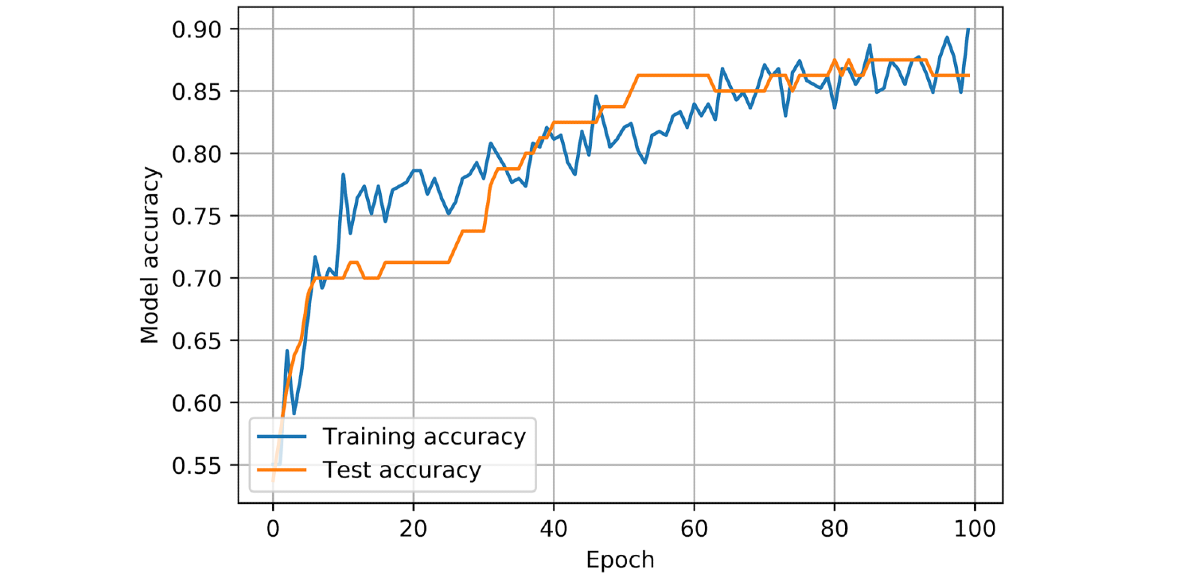
Model accuracy of an artificial neural network prognostic model for coronavirus disease (COVID-19) in a UK population. Model accuracy is based on training and test set results per epoch. Accuracy is defined as (TP+TN)/(TP+TN+FP+FN), where TP=true positive, TN=true negative, FP=false positive, and FN=false negative. One epoch represents one full cycle through the training set data. As the model trains for a greater number of epochs, the accuracy (ie, its ability to predict true positives and true negatives relative to all outcomes) increases.

**Figure 3:**
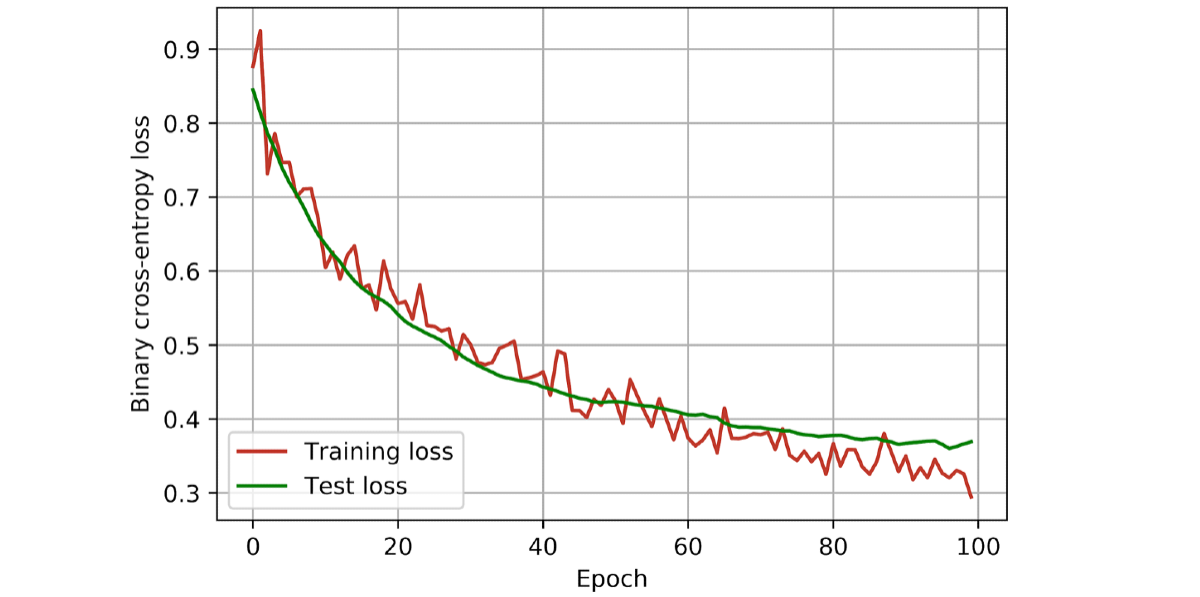
Performance metric of an artificial neural network prognostic model for coronavirus disease (COVID-19) in a UK population. Binary cross-entropy loss for training and tests per epoch. Cross-entropy loss measures the performance of a model that outputs a prediction between 0 and 1. It is a measure of how far the predictions made by the model are from the truth. As loss decreases, the probabilities estimated by the model match the actual target value (in this case, correct mortality predictions) more closely.

**Figure 4:**
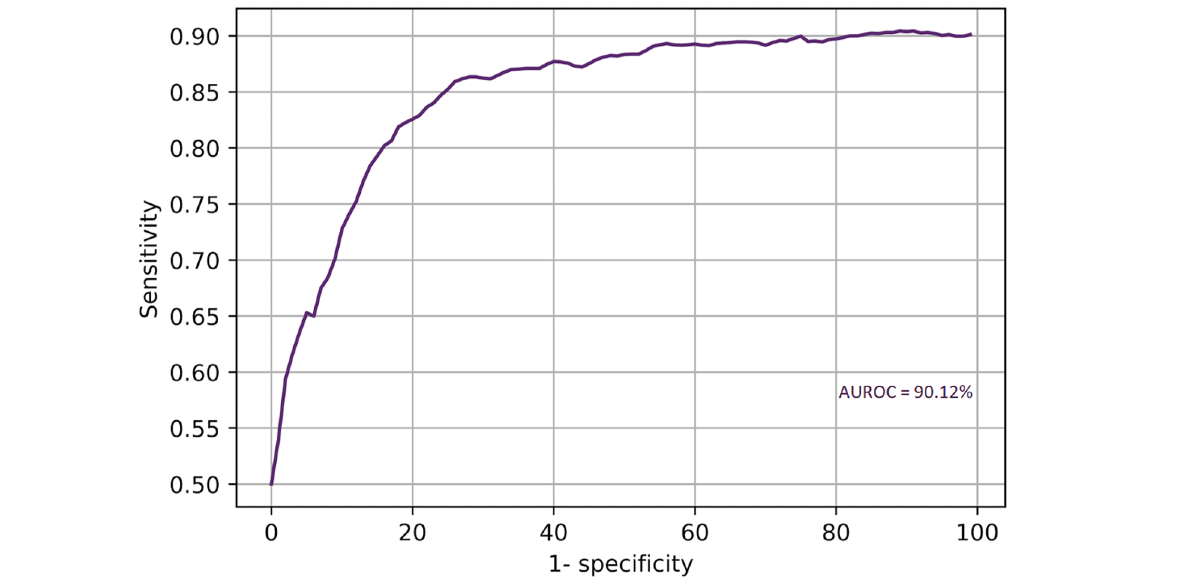
Receiver operating characteristic curve for an artificial neural network prognostic model for severe acute respiratory syndrome coronavirus 2 (SARS-CoV-2) in a UK population. AUROC: area under the receiver operating characteristic curve.

With this ANN, cross-validated accuracy (accuracy on the training and validation set) was 89% (95% CI 81%-97%). Patient-specific mortality was predicted with 86.25% accuracy on the test set (Figure 2), with a sensitivity of 87.50% (95% CI 61.65%-98.45%) and specificity of 85.94% (95% CI 74.98%-93.36%). The positive predictive value was 60.87% (95% CI 45.23%-74.56%), and the negative predictive value was 96.49% (95% CI 88.23%-99.02%). Binary cross-entropy loss is demonstrated in Figure 3. The AUROC was 90.12% (Figure 4).

### Relative Importance of Clinical Attributes

**Figure 5:**
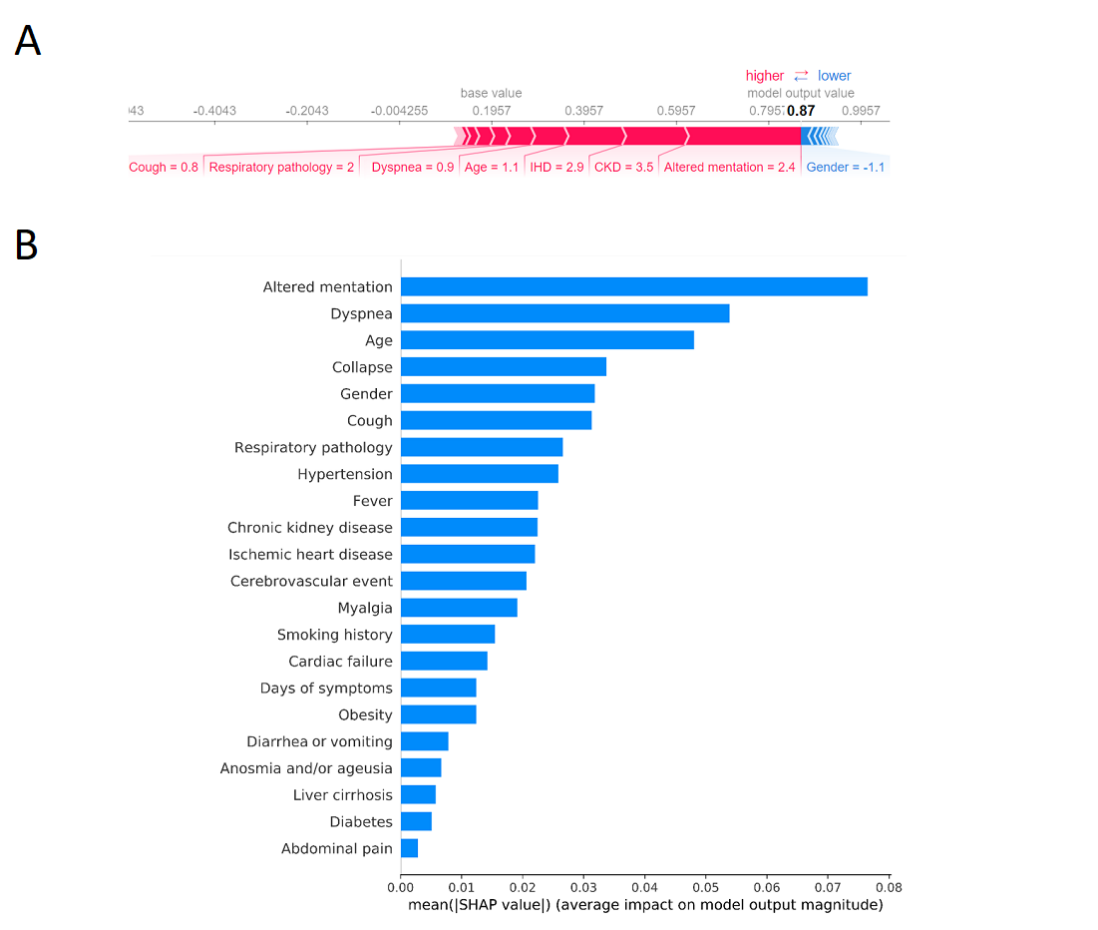
Relative importance of clinical attributes in an artificial neural network prognostic model for severe acute respiratory syndrome coronavirus 2 (SARS-CoV-2) in a UK population. A) An example of an accurate mortality prediction. Feature importance is proportional to feature bar width. Features which increased risk of death are shown in red, and those which decreased risk are shown in blue. Altered mentation, ischemic heart disease (IHD), and chronic kidney disease (CKD) were the most salient predictors of mortality for this patient. Female gender was protective. B) Overall feature importance as considered by the model. SHAP: Shapley additive explanations.

The approximation algorithm for SHAP values could reveal feature importance on a patient-specific basis (Figure 5A). The model dynamically adjusts mortality risk prediction for each patient, and illustrates the predictors it used (and their relative importance) to form the prediction. Overall feature importance for the model was also calculated (Figure 5B). Altered mentation, new dyspnea, and increasing age were the most significant predictors of mortality. Moderate predictors of mortality were collapse, male gender, new cough, and known respiratory pathology.

## Discussion

### Principal Findings

In this analysis, we provide details of an ANN capable of predicting patient-specific mortality with high sensitivity and specificity. Furthermore, the model provides information on which features are most salient when predicting risk, delivering explainable predictions on a patient-specific basis to clinicians and potentially allowing more informed discussions with patients and relatives.

Our aim was to provide a patient-specific, point-of-admission mortality risk prediction to help inform clinical management decisions at the earliest opportunity. The contribution of this analysis is in the proof-of-concept ANN trained on data from a single site, which demonstrates the early utility of deep learning approaches in a rapidly evolving pandemic with no established or validated prognostic scoring systems. Intensivists and respiratory physicians can be alerted of a patient with a higher relative risk of deterioration at an earlier stage, and ITU departments can better anticipate bed needs and adjust staffing and capacity appropriately.

Altered mentation, dyspnea, and increasing age were found to be the most salient overall features in predicting mortality. Moderate predictors of mortality included collapse, male gender, new cough, and previous respiratory pathology. These features are broadly in line with the current literature [31].

Of note is smoking history, which appears less important to the model than might be intuitively assumed. Smoking history was encoded as a presence feature, meaning that current smokers were grouped with ex-smokers, and this may provide an explanation as to why smoking history was considered as a more minor feature by the ANN. Indeed, the largest study to investigate factors associated with SARS-CoV-2 deaths to date (n=5683 SARS-CoV-2–linked deaths) demonstrated a lower risk of death in current smokers (hazard ratio 0.88; 0.79-0.99) but a higher risk in ex-smokers (hazard ratio 1.25; 1.18-1.33) [33]. Although this may suggest smokers are underrepresented in groups with severe disease, a protective mechanism related to nicotine function has been suggested [34].

An advantage of our current model is that all demographic, comorbidity, lifestyle, and symptom data can be collected on first encounter with a physician, and therefore an early outcome prediction can be produced following clerking. Unlike previous work [11,12], the intended use of this prognostic model as a point-of-admission mortality risk predictor is clearly described. Furthermore, the ANN models outcomes in the context of current SARS-CoV-2 practice guidelines and is therefore directly applicable to the current cohort of hospitalized patients with confirmed SARS-CoV-2.

There were several limitations to consider in our analysis. Although the ANN is representative of hospitalized patients with confirmed SARS-CoV-2 and their outcomes within the geographic remit of the study site, these results should be generalized with caution to other populations. Validating the predictive ability of the model would require prospective studies with a larger number of patients across multiple sites in the United Kingdom. In addition, we were unable to account for patients who were admitted for clinically suspected SARS-CoV-2 and subsequently tested positive for the virus, but who may have died due to an unrelated morbidity. However, such patients likely represent a small minority of our cohort. There were 9 patients (2.2% of our data set) who were alive at the end of the study period but had not yet been discharged. They were considered as alive in the analysis. However, these censored patients may be more likely to have a favorable outcome, as we found that the mean length of stay for patients who died was shorter relative to the whole cohort. Mean length of stay for all patients was 9.8 days, and therefore the ANN would be unsuitable for use to predict outcomes for significantly longer durations than this. Lastly, the current model does not include other potentially important predictors of outcomes, including hematological, biochemical, radiological, microbiological, and histological results where appropriate. The ANN architecture is such that adding further input variables (including clinical investigation results) is easily achievable. We plan to extend the ANN in the future with these parameters to maximize its predictive capability.

More complex deep learning models such as recurrent neural networks (RNNs) allow for time-series forecasting and have been successfully used to predict outcomes in real time [17]. Use of RNNs in the future would allow for real-time outcomes predictions throughout an individual admission and could account for factors such as ITU admissions as they occur.

### Conclusions

Increasingly, hospitals document and store patient data in EHRs, and machine learning techniques are becoming more ubiquitous in health care. In the context of an evolving pandemic with no established prognostic scoring system, deep learning approaches can be used to rapidly develop empirical prognostic models. These models have the inherent advantage of becoming progressively more accurate and representative as data sets increase in size. With larger, more representative data sets and more accurate artificial intelligence models, it may be possible for patient-specific outcome predictions to help guide physicians to tailor management and establish appropriate ceilings of care more generally.

## Abbreviations

ANN: artificial neural network
AUROC: area under the receiver operating characteristic curve
COVID-19: coronavirus disease
EHR: electronic health record
RNN: recurrent neural network
SARS-CoV-2: severe acute respiratory syndrome coronavirus 2
SHAP: Shapley additive explanations
